# Integral Sensor Fault Detection and Isolation for Railway Traction Drive

**DOI:** 10.3390/s18051543

**Published:** 2018-05-13

**Authors:** Fernando Garramiola, Jon del Olmo, Javier Poza, Patxi Madina, Gaizka Almandoz

**Affiliations:** Faculty of Engineering, Mondragon Unibertsitatea, 20500 Arrasate - Mondragón, Spain; jdelolmo@mondragon.edu (J.d.O.); jpoza@mondragon.edu (J.P.); pmadina@mondragon.edu (P.M.); galmandoz@mondragon.edu (G.A.)

**Keywords:** sensor fault diagnosis, diagnostic observer, fault injection, railway traction drive, frequency analysis

## Abstract

Due to the increasing importance of reliability and availability of electric traction drives in Railway applications, early detection of faults has become an important key for Railway traction drive manufacturers. Sensor faults are important sources of failures. Among the different fault diagnosis approaches, in this article an integral diagnosis strategy for sensors in traction drives is presented. Such strategy is composed of an observer-based approach for direct current (DC)-link voltage and catenary current sensors, a frequency analysis approach for motor current phase sensors and a hardware redundancy solution for speed sensors. None of them requires any hardware change requirement in the actual traction drive. All the fault detection and isolation approaches have been validated in a Hardware-in-the-loop platform comprising a Real Time Simulator and a commercial Traction Control Unit for a tram. In comparison to safety-critical systems in Aerospace applications, Railway applications do not need instantaneous detection, and the diagnosis is validated in a short time period for reliable decision. Combining the different approaches and existing hardware redundancy, an integral fault diagnosis solution is provided, to detect and isolate faults in all the sensors installed in the traction drive.

## 1. Introduction

In the last few decades, electric drives have become more important with the increase of machinery electrification and electric vehicles. Moreover, in Railway applications, the availability of the traction drive is directly linked to the availability of the complete system, as a train could stop in case of a failure in the traction drive. Maintenance activities have an important influence on the availability of the system, being an ideal maintenance the one which prevents a failure [1], based on the health of the system. A Fault Diagnosis is needed in order to detect faults and implement a Condition-Based Maintenance.

Fault diagnosis functionalities or tools can differentiate between some industrial applications and companies from others. With fault diagnosis, we refer to the sequence of actions needed to detect, locate, and identify the fault mode in a system. Moreover, the severity of the fault can be obtained. This is known in the literature as Fault Detection and Diagnosis (FDD) [2,3]. If the fault is only being detected and located, the approach is called Fault Detection and Isolation (FDI) [4]. In this case, the specific fault mode or the severity is not established.

Different FDI approaches have been presented in the literature. In [5], a classification between model-based and model free approaches is done. Quantitative model-based FDI approaches, also referred as Model-based FDI, are based on an analytical redundancy, so the measured value is compared to a value obtained from the model. The difference between both values is called residual, and it should be close to zero in fault-free cases. A comparison among the different Model-based FDI approaches is presented in [6].

In this research, on-board Model-based FDI approaches are proposed and implemented in a commercial Traction Control Unit (TCU) for a tram. In complex systems, a fault can concern several signals, being a Model-Based FDI a suitable solution to improve detection sensitivity [7]. The model of the traction drive is already available, as it is defined at the beginning of the design phase for performance simulation, so a Model-based FDI can be validated during this period too. In addition, the design of FDI approaches based on models can benefit from the knowledge and models gathered during the common design phase of the system.

On the other hand, as model-free approaches need a large quantity of historical data, on-board diagnosis has limitation due to computational requirements [8]. On-board diagnosis is closer to physical systems, improving the diagnosis celerity and reducing the data communications costs. Moreover, in moving systems, the communication to remote diagnosis cannot be executed at high frequencies and on-board data storage is limited. Thus, this research is focused on Model-based FDI approaches implemented in a commercial TCU for a tram.

Once a fault occurs, system performance deteriorates from the nominal zone to the degraded zone. Thus, a Model-based FDI for early detection of faults in traction drive elements, before the system passes from degraded mode to failure, is an important point to increase the availability and reliability of the system. The types of faults in traction drives can be classified as sensor, actuator, and process faults [9]. Fault Detection and Isolation (FDI) approaches have been implemented in electric drives, mainly for sensors [10,11], electric machines [12,13] and power converters [14,15]. Traction control strategy needs the sensor feedback for properly operation, so a faulty sensor can suppose a loss of availability and performance deterioration [16]. This research is focused on sensor fault detection and isolation in a traction drive.

In [9] a review of FDI methods for sensor faults in aerospace applications is presented. Recently, an integrated diagnosis for aerospace application was presented in [17], which includes a sensor fault diagnosis and performance degradation estimation. There are several recent publications in sensor diagnosis for different applications in electric and hybrid vehicles [18,19,20], mainly in order to increase safety and availability. However, the publications in Railway systems are reduced and limited to test benches without commercial control units, observer-based FDI for sensor faults are proposed in [2,21,22].

The aim of this article is to propose an integral FDI solution for sensors in a Railway Traction drive, based on different FDI approaches. The applied approaches do not imply any hardware change. Railway applications are not safety-critical systems as aerospace systems. In aerospace applications, it is critical to react instantaneously to the fault, in order to activate a fault tolerant solution. On the other hand, in Railway applications, the control system can stop and restart the traction unit in seconds maintaining the motion of the train. Thus, the proposed approaches in this article for Railway application should be evaluated during a short time period in order to confirm the fault detection.

The most suitable FDI approach for each sensor has been selected, based on the following factors: algorithm complexity, hardware and software resources available in the traction drive, tuning difficulty due to parameter variation/uncertainties and reliability. A simple model for an observer, avoiding several motor parameter variation during operation [23], the available hardware redundancy and low computational algorithms, which can be executed in the TCU without demanding an increase of the execution period, are the preferred choices.

Among the FDI solutions for DC-link voltage and catenary current sensor, an observer-based FDI approach based on the input filter is proposed. In [21], a similar solution is presented, as an adequate solution for real time implementation, which avoids problems in the modelling of the power converter or the need of a FPGA-based FDI [24], allowing an easier implementation in the Digital Signal Processor available in the TCU. In [25], an Extended Kalman Filter is implemented for FDI, based on induction motor model. The author concludes that the performance of the approach deteriorates at very low speeds and it is affected by parameter variation. The proposed solution in this article does not require demanding hardware and software resources for real time implementation, due to the simplicity of the model of the input filter, in comparison to models including a power converter or an electric machine. Furthermore, among the observer-based approaches, Luenberger observer could be more adequate for industrial applications due to the possibility to simplify its algorithms [26]. In conclusion, the proposed solution is justified due to lower algorithm complexity, lower parameter variation and uncertainties of the input filter in comparison to more complex motor model, capability of the TCU and reliability of Luenberger observer.

In the Railway Traction drive studied, only two phase measurements are available. Thus, low computational cost FDI approaches for phase current sensor, such as those based on the sum of three current measurements cannot be applied [27]. There are few studies with just two phase measurements which analyze offset and gain faults, as is summarized in [28]. This work proposes a bank of observers for an induction motor based drive, using a Sliding Mode Observer and a High Gain Observer. In [29] a Sliding Mode observer is proposed for phase current sensor fault reconstruction for a Permanent Magnet Synchronous motor based drive. Both research works use the motor model and they assume that motor parameters are known and constants. On the other hand, in [30], a compensation of the oscillation generated due to phase current sensor fault is presented. Moreover, the frequency of the oscillation allows to the ability to distinguish between offset and gain fault. The extraction of the oscillation can be done by applying to current components $i_{d}$ and $i_{q}$, a low pass filter and a passband filter. The passband filter will be centered in the fundamental frequency of motor stator current for offset faults, or at twice this frequency for gain faults. Despite the limitations of this approach, as it is not possible to isolate faults between both phase current sensors, it allows to reutilize filters and control strategy algorithm already implemented in the traction control unit. Thus, it requires lower computational resources compared to the aforementioned observer-based approaches. On the other hand, as it does not depend on the motor model, it does not need an online motor parameter adaptation to avoid performance deterioration, as these parameters change during operation [23]. In conclusion, the proposed solution is considered the most suitable for the Railway Traction drive, due to lower algorithm complexity in comparison to a bank of observers or a Kalman filter, the reutilization of available control and filter algorithms, capability of the TCU, and the reliability of the detection.

Finally, in case of the speed sensor, due to the hardware redundancy already available in the Railway Traction drive configuration, observer-based FDI approaches for speed sensor faults [31], have not been implemented. Mainly, due the reliability of the hardware redundancy, being the detection decoupled from parameter variation and uncertainties, as well as the low computational cost and TCU capability, the solution based on hardware redundancy has been proposed.

Previous works in sensor FDI in Railway applications have been validated in simulation [2,32] or in an experimental test bench [21,22], but without commercial TCU, and they do not include all the sensors installed in a railway traction drive.

In contrast to previous works, a commercial Railway control unit was used for Hardware-in-the-loop simulation (HIL). The HIL platform is composed of a Real Time Simulator and a Traction Control Unit (TCU) for a Railway application. The TCU is a commercial unit for a tram developed by CAF Power & Automation (Spain). Thus, the FDI algorithms were implemented together with the same control software utilized for a real tram application.

The paper has the following structure: Section 2 presents the Railway traction drive description and problem statement. Section 3 presents the integral sensor fault diagnosis structure. Section 4 proposes a FDI approach for DC-link voltage and catenary current sensors. Section 5 presents a FDI approach for motor phase current sensors. In Section 6, an approach for speed sensor based on hardware redundancy is presented. In Section 7 the validation in a HIL platform is presented. Finally, the discussion and conclusions are given.

## 2. Railway Traction Unit Description and FDI Strategy Objectives

**Figure 1:**
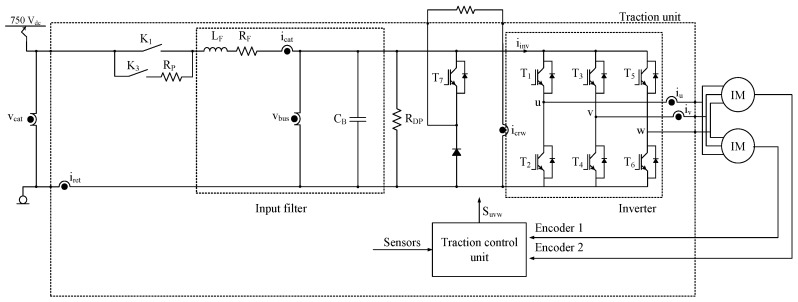
Railway traction unit.

There are different traction unit topologies but this research has been applied to the traction unit shown in Figure 1.

The traction unit is supplied from a 750 V DC catenary. The traction unit can be divided into:

- Main and pre-charge contactors, represented as K_1_ and K_3_.
- Input filter, composed of L_F_, C_B_ and R_F_, with functions being the catenary current ripple reduction, protection against catenary voltage changes, and a DC-link voltage setting.
- Braking unit given by IGBT T_7_ and braking resistor.
- IGBT-based inverter that supplies two induction motors in parallel.
- Traction control unit, where software for the control strategies, protections and alarms, is executed.

The list of sensors in the traction unit is given in Table 1. The objective of the integral supervision is to present FDI approaches based on hardware and analytical redundancy, without including additional sensors.

## 3. Integral Sensor Fault Diagnosis Structure

The proposed Integral Sensor Fault Diagnosis strategy provides the most suitable solution for each sensor, based on the architecture of the traction unit shown in Figure 1. In some cases, the addition of an additional sensor might be a new solution, more suitable, but it can have drawbacks too, due to new hardware or software requirements for the Traction control unit. Thus, approaches which provide analytical or hardware redundancy without hardware changes are proposed.

**Figure 2:**
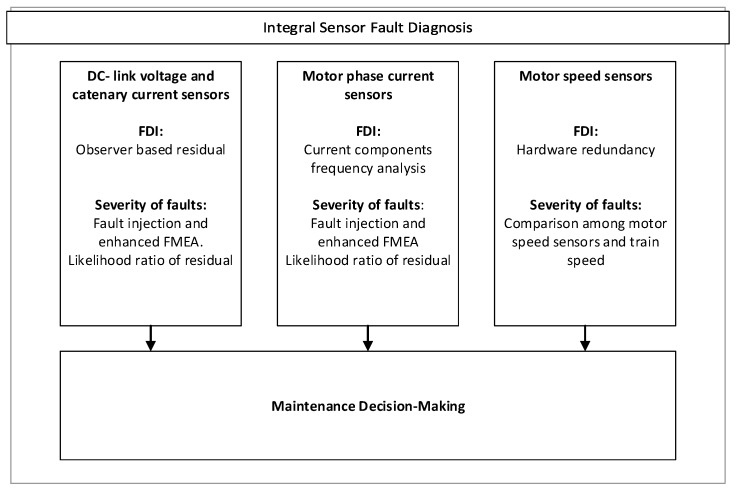
Integral Sensor Fault Diagnosis structure for traction unit.

Among the several FDI strategies developed for the traction drive, the ones presented in this article are shown in Figure 2, and explained in following sections. These strategies are centered in the FDI for DC-link voltage, catenary current, motor phase current and speed sensors. Solutions for the rest of sensor (catenary voltage, return current, and crowbar current) are not discussed here, as they are based on similar FDI approaches. Catenary voltage sensor faults can be isolated using hardware redundancy, since more than one sensor is installed in the train. Return current sensor faults are isolated using the redundancy with catenary current sensors. Finally, crowbar current sensor fault detection can be performed during braking with the observer based FDI approach presented in Section 4. With the combination of different FDI approaches, sensor faults can be isolated.

The different FDI strategies are executed in parallel. A suitable feedback gain selection for observer based FDI, makes residual for detecting catenary current sensor faults sensitivity low to DC-link voltage sensor faults, and residual for detecting DC-link voltage sensor faults sensitivity low to catenary current sensor faults. Thus, both residuals are decoupled.

In order to avoid any false detection, as observer based residual sensitivity depends on observer gains selection, a procedure is proposed to implement the integral diagnosis strategy for FDI in current and voltage sensors.

The estimation of fault severity is not deeply described in this article. In case of current and voltage sensors faults, a previous fault injection and analysis, for developing an enhanced Failure mode and effects analysis (FMEA) is needed [33]. This analysis links the amount of deviation of the sensor with the root failure mode. Then statistical tools as likelihood ratio are applied in order to estimate the fault severity.

## 4. FDI Approach for DC-Link Voltage Sensor and Catenary Current Sensor

Several FDI approaches were presented for DC-link sensors. In [34], a comparison between power and estimated motor input power is used for DC-link sensor fault FDI. This method has limitations for low speed and it depends on the stator resistance and inverter losses estimation. In [25] an Extended Kalman filter is proposed for DC-link voltage sensor FDI. This method is based on the motor model, which needs accurate parameter configuration and has high computational costs. On the other hand, [21] proposes an Observer-based FDI method, which is not dependent on the motor model. It is based on a Luenberger observer [35] applied to a single phase PWM rectifier input filter. These kind of strategies has the advantage of modelling the input LC filter of the traction drive, which has a linear model, based on manageable first order differential equations. In [22], a Sliding mode observer (SMO) is proposed instead of a linear gain observer.

Taking into account the solutions presented in the literature and their advantages and drawbacks, a FDI approach that is independent of motor parameters and based on the input filter of the traction unit was proposed.

### 4.1. Input Filter System Model

Similar to previous publications [36], the model of the input filter in state space is presented in (1), being $x^{T}=\left[\begin{array}{cc} i_{cat} & v_{bus} \end{array}\right]$, $u^{T}=\left[\begin{array}{ccc} v_{cat} & i_{inv} & i_{crw} \end{array}\right]$ and $y^{T}=\left[\begin{array}{cc} i_{cat} & v_{bus} \end{array}\right]$. The $i_{inv}$ value is not directly measured, but it is calculated from T1, T3, and T5 switches states and $i_{u}$ and $i_{v}$ current sensors measurements.

(1) $$\begin{array}{c} \frac{dx}{\mathrm{dt}}=\left[\begin{array}{ll} -\frac{R_{F}}{L_{F}} & -\frac{1}{L_{F}}\\ \frac{1}{C_{B}} & 0 \end{array}\right]x+\left[\begin{array}{ccc} \frac{1}{L_{F}} & 0 & 0\\ 0 & -\frac{1}{C_{B}} & -\frac{1}{C_{B}} \end{array}\right]u\\ y=\left[\begin{array}{cc} 1 & 0\\ 0 & 1 \end{array}\right]x. \end{array}$$

Before designing the observers, the observability and controllability of the system, given by (1) was checked. The controllability for a linear system is given if Expression (2) was fulfilled, n being the dimension of the state vector x. The rank obtained was 2, so it can be concluded that the system is fully controllable.

(2) rank(B ⋮ AB)=n

The next step was to check the observability of the system, given if Expression (3) is fulfilled. The rank is 2, so it can conclude that the system is fully observable.

(3) $$rank\left(\begin{array}{c} C\\ \cdots \\ AC \end{array}\right)=n$$

Based on [37], the detectability and isolability analysis was done for the configuration represented in (1). It was assumed that the fault modes were additive and constant during the time window. In Table 2, the x in the detectable column represents that the fault mode was detectable. The x in each fault column represents that the fault is isolable respect to each row, whereas the 0 represents that the fault mode is not isolatable.

Thus, from the results presented in Table 2, it can be concluded for example that it is not possible to isolate an additive fault in sensor $v_{bus}$ from an additive fault in sensor $v_{cat}$. The same happens for isolating a fault in sensor $i_{crw}$ from a fault in $i_{inv}$, or isolating a fault in sensor $v_{bus}$ from a fault in sensor $i_{cat}$ without any other redundancy apart from the system given in state space representation (1).

In the following subsection, a FDI approach based on a bank of observers for $v_{bus}$ and $i_{cat}$ was proposed [36], providing analytical redundancy to solve one of the previously mentioned isolation problems. Although the diagnosability analysis has been done for additive fault modes, in the following FDI strategy both fault modes, additive and multiplicative, will be analysed, as offset and gain faults will be injected.

### 4.2. FDI Strategy for DC-Link Voltage and Catenary Current Sensors

In order to isolate DC-Link voltage sensor faults from catenary current sensor faults, a bank of two Luenberger observers was proposed. Observers are based on the model of input filter system in (1), and in consequence, the strategy is independent from the motor model. The input filter model is simpler than the motor model and it has fewer uncertainties and parameters, so the observer implementation was easier and it had less computational requirements.

A different feedback strategy for each observer is used depending on the fault that is being detected. If the detection is focused on DC-Link sensor faults, the observer equations for DC-link voltage are given in (4), for $C_{1}=\left[1\;0\right]$ and $y_{1}=i_{cat}$.

(4) $$\begin{array}{c} \dot{\hat{x}}\left(t\right)=A\hat{x}\left(t\right)+Bu\left(t\right)+L\left(y_{1}\left(t\right)-C_{1}\hat{x}\left(t\right)\right)\\ {\hat{y}}_{1}\left(t\right)=C_{1}\hat{x}\left(t\right). \end{array}$$

**Figure 3:**
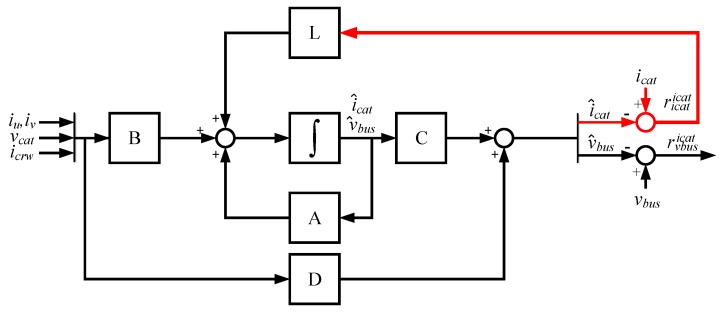
Diagnostic observer for DC link voltage sensor FDI.

In Figure 3, the observer model for DC-link voltage sensor FDI is presented. As it can be seen in (4), the observer does not take into account the $v_{bus}$ measured, so the ${\hat{v}}_{bus}$ estimated is not influenced by the DC-link voltage sensor fault. Thus, in the case of a faulty $v_{bus}$ sensor, the fault will be detected and isolated in the residual $r_{vbus}^{icat}={\hat{v}}_{bus}-v_{bus}$. On the other hand, the observer estimation is influenced by a $v_{cat}$ sensor fault, so hardware redundancy of this sensor, available in distributed railway traction configurations in the train should be used to discard the $v_{cat}$ sensor fault.

The system dynamic is given by the poles obtained solving the equation presented in (5). Normally the closed loop poles are fixed to be between three and six times faster than the open loop poles [22]. Higher dynamics make the observer more sensitive to measurement noises. Thus, the L gain matrix is obtained with the poles placement method. Closed loop poles have been chosen to be five times faster than open loop poles.

(5) |sI−(A+LC)|=0.

**Figure 4:**
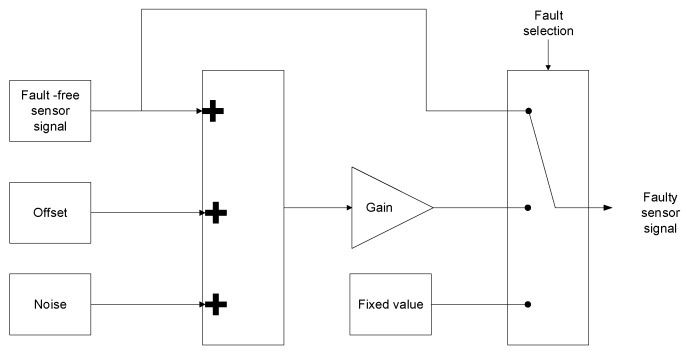
Sensor fault injection.

The traction system and the sensor faults injection blocks have been modelled in Matlab-Simulink. Based on the most common sensor fault modes [9] and information from the CAF Power & Automation maintenance team, gain (scaling), and offset (bias and drift) faults have been modelled, as shown in Figure 4. Disconnection faults were not considered, since the drive protection system shuts itself down as quickly as possible, when overcurrents and overvoltages, due to hard faults, are detected. The available time interval between the fault occurrence and system shut down does not allow for any FDI task execution. The fault injection model allows injecting different sensor fault modes easily and quickly. Fault injection has been previously used in electric drives applications [38]. The aim of FDI in this work was early detection, before the system passes from degraded zone to failure.

**Figure 5:**
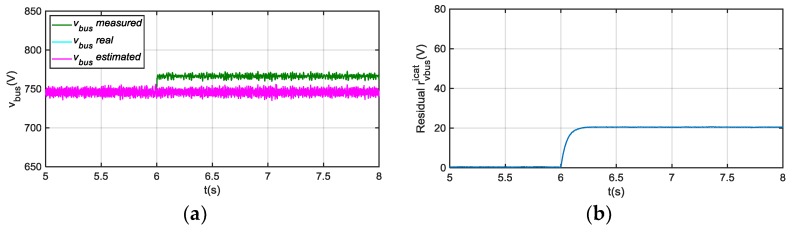
(**a**) Direct current (DC)-link voltage for 20 V offset fault injection in $v_{bus}$ sensor at 6 s; (**b**) $\left|r_{vbus}^{icat}\right|$ filtered from difference between measured and estimated $v_{bus}$.

**Figure 6:**
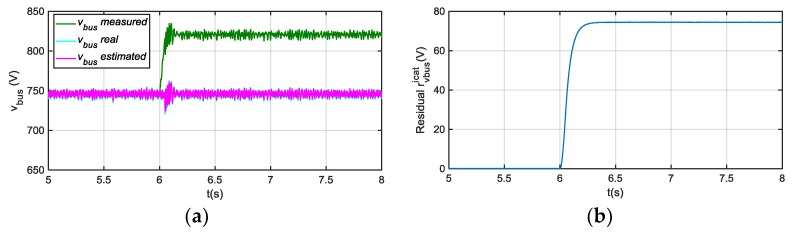
(**a**) DC-link voltage for +10% gain fault injection in $v_{bus}$ sensor at 6 s; (**b**) $\left|r_{vbus}^{icat}\right|$ filtered from difference between measured and estimated $v_{bus}$.

The real $v_{bus}$, obtained from the modelled system, the sensor measured $v_{bus}$, and the observer estimated ${\hat{v}}_{bus}$ are displayed for an offset sensor fault in Figure 5 and gain sensor fault in Figure 6. Despite the faulty measurement of $v_{bus}$ sensor, the estimated ${\hat{v}}_{bus}$ follows the real value, as the estimation does not depend on this sensor. Furthermore, the filtered residual $\left|r_{vbus}^{icat}\right|$ is shown too. Residual $r_{vbus}^{icat}$ is obtained from comparison of $v_{bus}$ measurement and estimated ${\hat{v}}_{bus}$. Thus, the increase of the residual can be seen when offset or gain faults are injected in $v_{bus}$ sensor, because the estimated value is decoupled from $v_{bus}$ sensor measurement, as measured value is not use for feedback loop, so the faulty sensor is easily detected.

Once, the fault is detected, the isolability of the sensor is analysed. It has to be taken into account that a faulty DC-Link voltage sensor is not the only one that can change the value of $r_{vbus}^{icat}$. Different fault modes are injected in other sensors in the system, and the residual $r_{vbus}^{icat}$ is monitored. Thus, an offset fault in phase current sensor $i_{u}$, generates an oscillation in system variables, but the estimated ${\hat{v}}_{bus}$ keeps on following the real value, and the effect on the average residual is negligible compared to faulty $v_{bus}$ sensor measurement.

**Figure 7:**
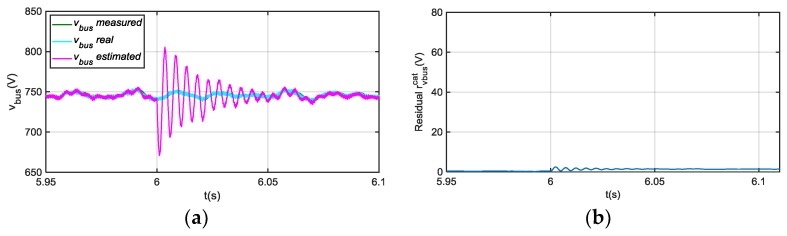
(**a**) Measured, real and estimated $v_{bus}$ for 20 A offset fault injection in $i_{cat}$ sensor at 6 s; (**b**) $\left|r_{vbus}^{icat}\right|$ filtered from difference between measured and estimated $v_{bus}$.

In case of an offset fault injection in sensor $i_{cat}$, an oscillation arises during a transient, as it is shown in Figure 7, but after 50 ms, estimated ${\hat{v}}_{bus}$ follows the real value. The effect on the residual $r_{vbus}^{icat}$ is low, compared to a $v_{bus}$ sensor fault, so a suitable threshold can avoid false $v_{bus}$ sensor fault detection and isolation. Furthermore, the fault detection and isolation decision is taken after the residual overpasses the threshold continuously during a short time period, so this allows filtering transient values in the residual.

**Figure 8:**
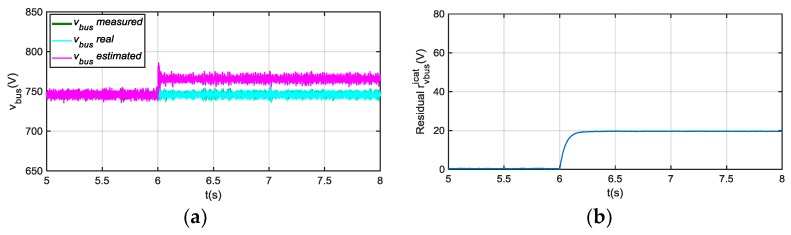
(**a**) DC-link voltage for 20 V offset fault injection in $v_{cat}$ sensor at 6 s; (**b**) $\left|r_{vbus}^{icat}\right|$ filtered from difference between measured and estimated $v_{bus}$.

**Figure 9:**
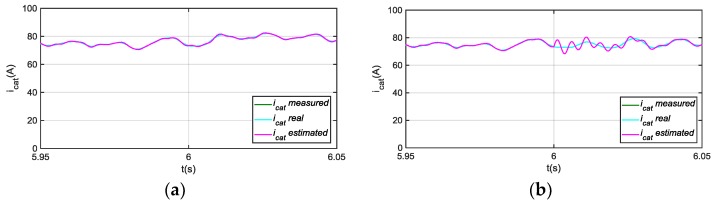
(**a**) Catenary current for 20 V offset fault injection in $v_{bus}$ sensor at 6 s; (**b**) Catenary current for 20 V offset fault injection in $v_{cat}$ sensor at 6 s.

The only shortcoming of this approach occurs when a $v_{cat}$ sensor fault needs to be detected and isolated. The effect in the estimated ${\hat{v}}_{bus}$ and$\;r_{vbus}^{icat}$, shown in Figure 8, is similar to the one generated by a $v_{bus}$ sensor fault. In this case, information coming from other traction drives in the distributed railway traction system should be analysed, to avoid wrong decisions. In addition to this information, in case of $v_{cat}$ sensor fault, a transient in the ${\hat{i}}_{cat}$ arises, which does not occur in case of $v_{bus}$ sensor fault, as it is shown in Figure 9. The faulty sensor logic decision is taken when the residual overpasses the threshold, which should be above it permanently during a predefined time. This produces a delay in the detection, which is not so critical in this application, but decreases the false detection risk due to abrupt changes in the residuals, produced by measurement noises.

Based on the residual obtained during fault injection in steady state, and the likelihood ratio calculation, it is possible to estimate the fault severity. Thus, in Table 3, the likelihood ratio obtained for different offset fault injection in $v_{bus}$ is presented. The likelihood ratio has been calculated offline, and for a 0.5 s time interval in steady state. The likelihood ratio is represented as s, being the subscript the fault free reference, and the superscript the different faulty cases. A negative likelihood ratio indicates that the fault free case is more probable, whereas a positive value indicates that the faulty case is more probable. The higher the value is, the more probable is the case.

Similar to the DC-link voltage sensor, a second Luenberger observer can be proposed for $i_{cat}$ sensor fault detection, as shown in (6), being $C_{2}=\left[0\;1\right]$, $x^{T}=\left[\begin{array}{cc} i_{cat} & v_{bus} \end{array}\right]$ and $y_{2}=v_{bus}$.

(6) $$\begin{array}{c} \dot{\hat{x}}\left(t\right)=A\hat{x}\left(t\right)+Bu\left(t\right)+L\left(y_{2}\left(t\right)-C_{2}\hat{x}\left(t\right)\right)\\ \hat{y}\left(t\right)=C\hat{x}\left(t\right). \end{array}$$

**Figure 10:**
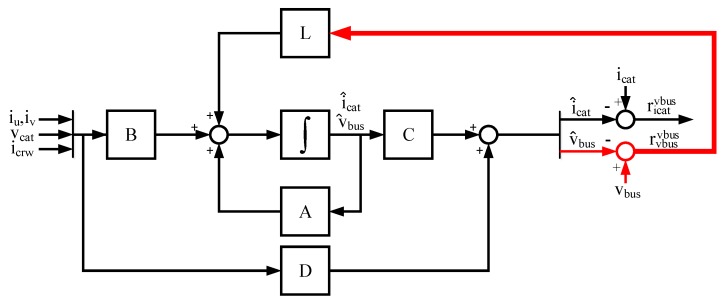
Diagnostic observer for catenary current sensor FDI.

**Figure 11:**
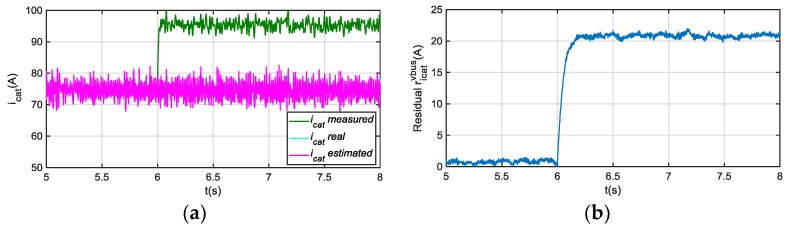
(**a**) Catenary current for 20 A offset fault injection in $i_{cat}$ at 6 s; (**b**) $\left|r_{icat}^{vbus}\right|$ filtered from difference between measured and estimated $i_{cat}$.

The observer model is presented in Figure 10. In this case, only the residual due to the difference between estimated and measured $v_{bus}$ is used for the feedback, so the ${\hat{i}}_{cat}$ estimation does not depend on the $i_{cat}$ sensor measurement. The residual $r_{icat}^{vbus}$ is used for FDI in current sensor $i_{cat}$, as shows Figure 11.

**Figure 12:**
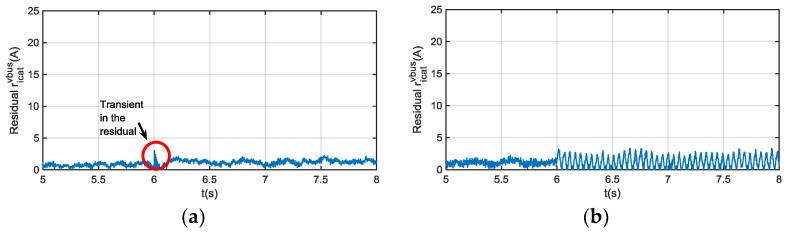
(**a**) $\left|r_{icat}^{vbus}\right|$ filtered for 20 V offset fault injection in $v_{bus}$ sensor at 6 s; (**b**) $\left|r_{icat}^{vbus}\right|$ filtered for 20 A offset fault injection in $i_{u}$ at 6 s.

Although, a transient in ${\hat{i}}_{cat}$ and $r_{icat}^{vbus}$ arises under$\;v_{bus}$ sensor fault, as it is shown in Figure 12, this residual sensitivity is low to $v_{bus}$ sensor faults in steady state. On the other hand, a ripple arises in the residual when a motor phase current sensor fault occurs, shown in Figure 12, so it can be used as additional information for phase current sensors FDI, which will be analysed in the following section. A fault in $i_{crw}$ sensor, is detected in $r_{icat}^{vbus}$, and it is easily isolated as it only occurs during braking. In conclusion, if no fault is detected in this residual during traction, but a fault is detected during braking, there is a fault in $i_{crw}$ sensor.

## 5. FDI Approach for Phase Current Sensors

With regard to phase current sensor FDI in electric drives, in [39] a bank of observers is proposed. Each observer has just one of the phase current sensors as input, so based on the estimation, it is possible the detection and isolation of faulty sensor. In contrast to this application, the system under study in this article uses only two phase current sensors, and the third current is calculated from the other two. Another bank of observers is proposed in [40] for a Double Fed Induction generator, normally used in wind turbines. In this case, only two phase currents are measured, but rotor current measurements are needed for stator current estimations, and stator current measurements for rotor current estimations.

On the other hand, in [40] a FDI approach based on the analysis of the probability density functions (pdf) of the sensor current signal is proposed. A phase current sensor fault generates a change in the pdf of $i_{d}$ current, obtained from the application of the Park transformation.

Finally, in [30,41] a compensation of the phase current sensor fault effect is proposed. Based on the frequency of the oscillations generated due to the sensor fault, it is possible to distinguish offset and gain faults. These approaches do not allow the fault isolation, but there are not dependent on motor model.

Based on the actual traction drive configuration, where only two phase current sensor are available and there is not any phase voltage sensor, the approach selected in this work was the analysis of the oscillations in the current components $i_{d}$ and $i_{q}$, generated by offset and gain faults. This approach is simple compared to other strategies, which are dependent on the model of the motor and parameter variability. Moreover, $i_{d}$ and $i_{q}$ are already calculated for the control strategy of the traction motor. The only shortcoming is that it is not possible to isolate the faulty phase current sensor, and both sensors should be checked to complete diagnosis.

**Figure 13:**
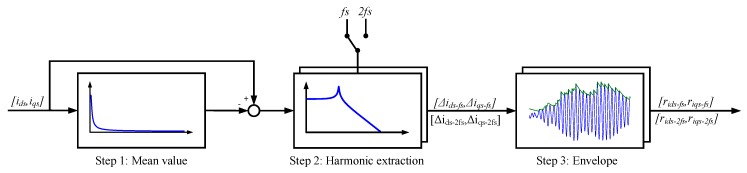
Current $i_{d}$ and $i_{q}$ components filtering for residual generation.

The residual generation process is divided into three different steps, as it is shown in Figure 13. The first step consists in eliminating the average value of the current components $i_{d}$ and $i_{q}$. An Exponential Smoother filter (ES) is used for this task [42]. The filter discrete transfer function is given by (7), being a=1−b. It is a recursive filter with an exponential ponderation, decreasing the influence of past samples as time goes by.

(7) $$H\left(z\right)=\frac{b}{1-az^{-1}}.$$

The second step is based on two passband filters [42], centered in $f_{s}$ and $2f_{s}$, being $f_{s}$ the fundamental frequency of motor stator current, which is obtained from flux and torque estimation. From previous analysis [30], it is known that offset deviations produce an additional oscillation in the current components $i_{d}$ and $i_{q}$, at $f_{s}$. Gain deviations generate the oscillation at $2f_{s}.$ Due to the oscillation generated, the first one allows to detect offset faults, whereas the second one detects gain faults. The discrete transfer function of the passband filter is given by (8), being $w_{0}$ and *b*, parameters to calculate in function of bandpass and sample frequency. Finally, the oscillation envelope is obtained in step 3.

(8) $$H\left(z\right)=\frac{\left(1-b\right)\left(1-z^{-2}\right)}{1-2bcos\left(w_{0}\right)z^{-1}+\left(2b-1\right)z^{-2}}.$$

**Figure 14:**
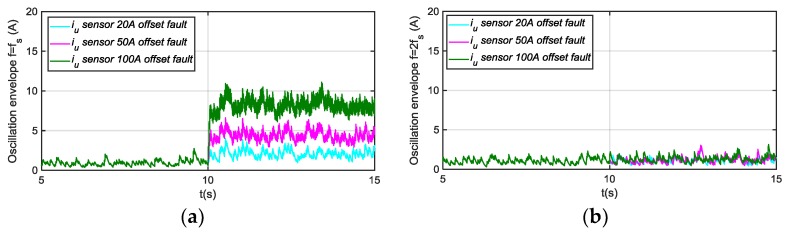
(**a**) Residual generated for offset faults injected in sensor $i_{u}$ and filtering frequency $f_{s}$; (**b**) Residual generated for offset faults injected in sensor $i_{u}$ and filtering frequency $2f_{s}$.

**Figure 15:**
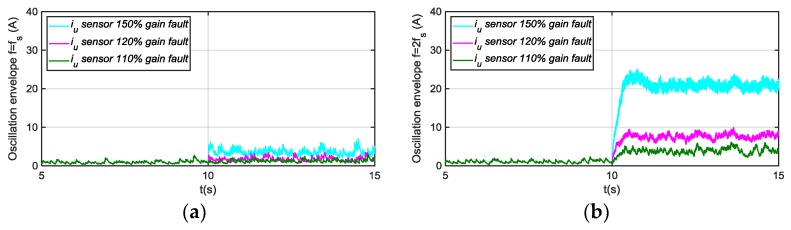
(**a**) Residual generated for gain faults injected in sensor $i_{u}$ and filtering frequency $f_{s}$; (**b**) Residual generated for gain faults injected in sensor $i_{u}$ and filtering frequency $2f_{s}$.

The residuals generated for different motor phase current sensor fault modes injection are shown next, being the references for torque and speed 600 Nm and 600 rpm, respectively. In Figure 14, the residuals based on a passband filter centered in $f_{s}$, and $2f_{s}$ for offset fault in sensor $i_{u}$ are presented. The residual based on a filter centered in $f_{s}$, is able to detect injected offset faults, whereas the residual based on $2f_{s}$ is not sensitive. As it is shown in Figure 15, the residual based on a passband filter centered in $2f_{s}$, is able to detect gain faults in $i_{u}$ sensor, whereas the one based on a filter centred in $f_{s}$, is not sensitive.

The fault severity estimation should be done as it was explained in previous sections, using information obtained from FMEA analysis and statistical tools.

## 6. FDI for Speed Sensor

Analytical redundancy for speed sensor diagnosis, based on observers, has been previously used in railway applications [31], but normally hardware redundancy for speed sensors is already available in distributed traction systems. Thus, based on the two speed sensor measurements available in the presented traction drive, and the average train speed calculated from different axes, a FDI algorithm was proposed.

**Figure 16:**
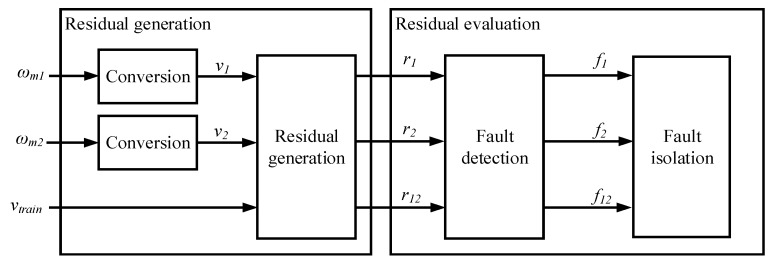
FDI for speed sensor.

The FDI structure is shown in Figure 16. The sensor fault detection and isolation is based on the difference among three linear speeds, two calculated form the encoders and the third one calculated as an average linear speed of all the distributed traction units. Thus, three different residuals (9) are proposed. Once, any of the residuals overpasses the threshold during an amount of successive samples, the corresponding logic indicator f is activated:

(9) $$\begin{array}{c} r_{12}=\left|v_{1}-v_{2}\right|\\ r_{1}=\left|v_{train}-v_{1}\right|\\ r_{2}=\left|v_{train}-v_{2}\right|. \end{array}$$

Depending on the combination of indicators, the faulty sensor is isolated. A relevant $v_{train}$ measurement deviation is not probable, as it depends on multiple sensor measurements, gathered from a variety of traction units. Anyway, if $f_{1}$ and $f_{2}$ indicators are given, it is recommendable to check the encoder sensors of another traction drive, in order to discard a multiple speed sensor fault (both encoders of one traction drive) at the same time. In Table 4, the combination of indicators for speed sensor isolation is shown.

## 7. Hardware-in-the-Loop Validation for FDI Approaches

**Figure 17:**
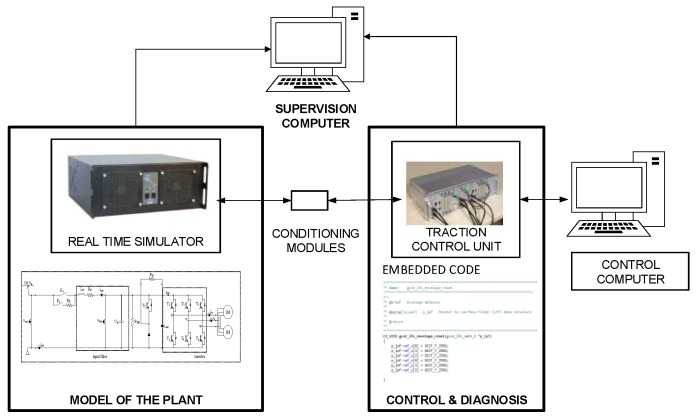
Hardware-in-the-loop platform.

The HIL platform used for validation is composed of a Real Time Simulator, from OPAL-RT Company, and a commercial Traction Control Unit, develop by CAF Power & Automation, for a Railway application, as it is shown in Figure 17.

The TCU is externally connected to the Real Time Simulator through analog and digital ports. Conditioning modules to adapt the inputs and outputs between TCU and Real Time Simulator are needed. This platform allows injecting faults, easily and quickly, in order to test the different FDI approaches.

The simulation step for model running in the Real Time Simulator is 15 µs. The TCU has a DSP for high speed execution. The sensor measurements are captured and monitored every 120 µs for validation purposes.

### 7.1. FDI Validation for DC-Link Voltage and Catenary Current Sensors

**Figure 18:**
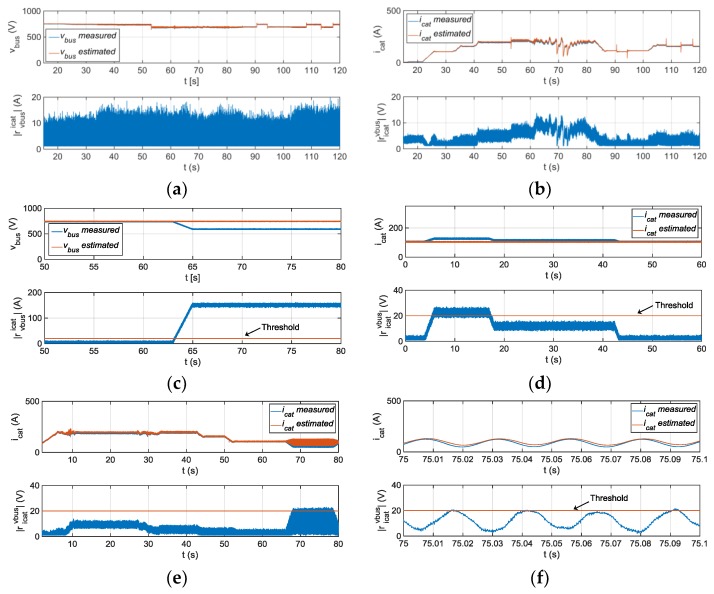
(**a**) Measured, and estimated $v_{bus}$ and $\left|r_{vbus}^{icat}\right|$ for fault-free operation; (**b**) Measured, and estimated $i_{cat}$ and $\left|r_{icat}^{vbus}\right|$ for fault-free operation; (**c**) Measured, and estimated $v_{bus}$ and $\left|r_{vbus}^{icat}\right|$ for fault injected in $v_{bus}$ sensor; (**d**) Measured, and estimated $i_{cat}$ and $\left|r_{icat}^{vbus}\right|$ for fault-injected in $i_{cat}$ sensor; (**e**) Measured, and estimated $i_{cat}$ and $\left|r_{icat}^{vbus}\right|$ for fault-injection in $i_{u}$ phase current sensor; (**f**) Oscillation generated in catenary current and $\left|r_{icat}^{vbus}\right|$ due to fault-injection in $i_{u}$ phase current sensor.

The Hardware-in-the-loop simulation results for DC-link voltage and catenary current sensors are shown in Figure 18. First, the residuals for normal operation are shown. The 𝓛∞ norm (10) is chosen for threshold setting, so residual thresholds should be higher than the maximum value of residual during normal operation. Then, the residuals $r_{icat}^{vbus}$ for FDI in catenary current sensor and $r_{vbus}^{icat}$ for FDI in DC-link voltage sensor are validated.

(10) $${\left\|u\right\|}_{\infty ,s}=\underset{i\in \left[k,k+s\right]}{sup}\left|u\left(k\right)\right|,$$

If the diagnostic observer dynamic is fast enough, only transients are appreciated in the residual $r_{vbus}^{vbus}$ and $r_{icat}^{icat}$. If slower dynamic is chosen, in order to increase the robustness to measurement noises, steady state error appears in $r_{vbus}^{vbus}$, so the threshold $r_{icat}^{vbus}$ should be increased to avoid false detections. Finally, it has to be taken into account that a phase current fault injection generates an oscillation in the catenary current and in the residual $r_{icat}^{vbus}$, which can generate a false alarm for catenary current sensor FDI, so the FDI for phase current sensors should be checked too, before taking decision. A low pass filter can be implemented too, in order to eliminate the oscillation in the residual.

### 7.2. FDI Validation for Phase Current Sensors

The FDI approach was implemented in the TCU and validated in the HIL platform. The following results are obtained for a torque reference of 600 Nm and motor speed of 600 rpm. The average value of the envelope depends on the operating point, so for a severity estimation of the fault, a previous relation between the envelope average value and the fault injected for different operating points should be obtained in HIL simulation. Thus, an adaptive threshold based on $i_{d,q}$ components, motor torque and speed are needed to estimated fault severity. In the case of fixed threshold, the sensibility of the FDI approach will be different depending on the operating point. For example, for a torque reference of 600 Nm, a threshold of 20 A will detect a +50% gain deviation, whereas the threshold needs to be decrease to 16 A, to detect the same fault for a torque reference of 200 Nm.

**Figure 19:**
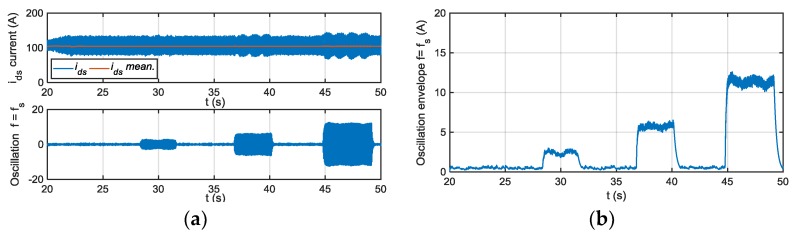
Residual generation for a +20 A, +50 A, and +100 A offset faults injected in phase current sensor, oscillation in (**a**) and envelope in (**b**).

In Figure 19 the results of deviations injected, as filtering steps are presented, oscillations in (a) and envelopes in (b). Different offset faults are injected into the $i_{u}$ sensor current. The current component average value, the oscillation filtered, generated due to the offset fault injected are shown. The $f_{s}$ centred filter extracts the oscillation due to an offset fault. Moreover, the envelope of the oscillation, which will be used as residual to compare to the threshold, is shown.

**Figure 20:**
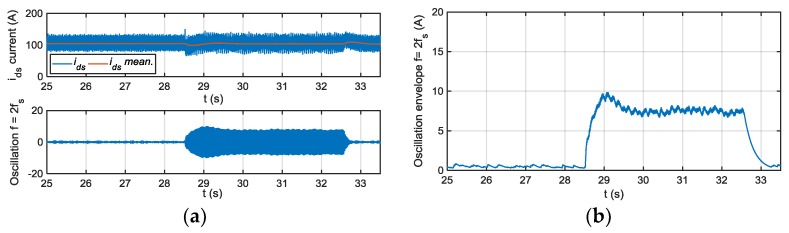
Residual generation for a 120% gain fault injected in phase current sensor, oscillation in (**a**) and envelope in (**b**).

In Figure 20, the different filtering steps for a gain fault injected in $i_{u}$ sensor current are presented, oscillation in (a) and envelope in (b). In this case, the oscillation is extracted by the $2f_{s}$ centred filter.

The sensibility of the FDI approach is better for low speeds, being higher in $i_{q}$ than in $i_{d}$ current component. On the other hand, $i_{q}$ is more sensitive to torque changes, so the filtering of this component can be more complicated. The FDI approach is able to differentiate between offset and gain fault modes, but it is not possible to isolate between faults in one phase or the other.

### 7.3. FDI Validation for Speed Sensors

**Figure 21:**
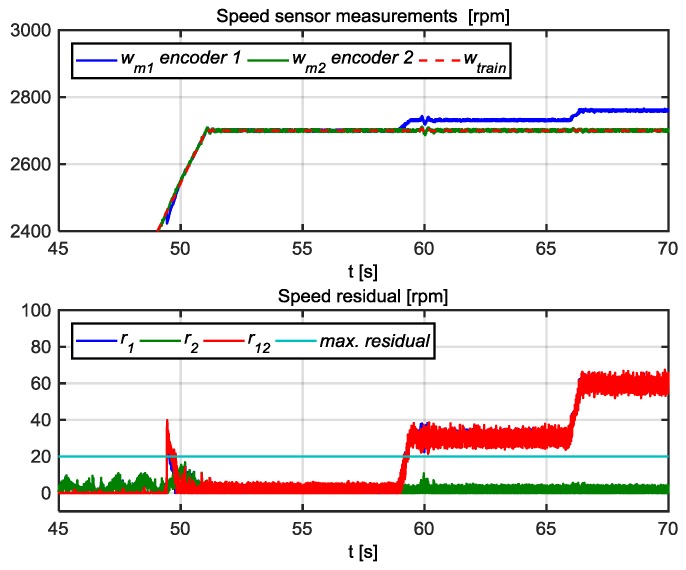
Speed measurements and speed residuals.

In Figure 21 the results for the FDI approach for speed sensors are shown. A gain fault corresponding to an increase of 27 rpm is set at 59 s and an increase of 54 rpm at 66 s in $w_{m1}$ speed sensor. In both cases, the residuals $r_{1}$ and $r_{12}$ overpass the thresholds, so the flags $f_{1}$ and $f_{12}$ will be activated. Based on Table 4, it can be concluded that the fault is in sensor $w_{m1}$. This FDI approach just analyses steady state residuals, whereas an anti-sliding algorithm processes transient differences among speed measurements. This kind of algorithms is commonly found in railway traction control systems.

## 8. Discussion

In this article, different FDI approaches have been presented to build an Integral Sensor Fault Detection and Isolation for a Railway traction drive. Furthermore, a proposal for the fault severity estimation has been presented too. An observer based FDI approach has been used for DC-link voltage and catenary current sensors, a signal analysis based FDI approach for phase current sensors and a hardware redundancy based FDI for speed sensors. Each approach has been justified as the most suitable one for the traction drive presented. The FDI approach selection has been done based on the following factors: algorithm complexity, hardware and software resources available in the traction drive, tuning difficulty due to parameter variation/uncertainties and reliability. The observer-based FDI for DC-link voltage and catenary current sensor uses the input filter model instead of the motor model. As input filter model is simpler, the influence of parameter variations and uncertainties is lower. Furthermore, a Luenberger observer is proposed, due to lower algorithm complexity in comparison to other solutions. The signal analysis based FDI for phase current sensors need low computational resources, as some algorithms are already available in the control strategy. Furthermore, as it is not based on a motor model, motor parameter estimation during operation is not needed. Finally, a redundant hardware based FDI is proposed for speed sensor faults, due to reliability and low computational cost.

Furthermore, the approaches developed in Matlab-Simulink have been simulated and implemented in a HIL platform with a real Railway TCU, designed for a tram. FDI approaches have been implemented in the DSP of the TCU, being the execution period 20 µs.

The presented fault severity calculation was not implemented in real time, in order to reduce the computational requirements for the DSP. The Integral Sensor Fault Detection and Isolation presented, allows detecting and isolating faults in all the sensors presented in the traction drive.

The FDI for the DC-link voltage and catenary current sensors is based on the input filter model of the traction drive, and it is not dependent on the motor model. The uncertainties and variability of parameters in the input filter are lower than in the motor model, which makes this solution easier to implement in a real application. Moreover, this FDI approach is not influenced by the operating point of the motor. The balance between the robustness and the sensitivity of the strategy is given by the observer feedback gain. Higher gains allow setting lower thresholds to increase the detection sensitivity, but this implies a lower robustness due to false alarms caused by measurement noises or other sensor faults. This work has set as a threshold of 20 A for current residual and 20 V for voltage residual, based on fault free behavior, so lower values should not be considered as a degraded zone. This FDI approach in combination with hardware redundancy, allows detecting and isolating faults in catenary current, crowbar current, return current, DC-link voltage and catenary voltage sensors. The fault severity estimation is calculated offline, based on previously obtained relations between the injected faults and generated residuals. Then a likelihood ratio is calculated with the residual values obtained in real time to estimate the most probable fault severity.

The FDI strategy for phase current sensors is able to detect two fault modes, offset and gain. Its main limitation is that, it is not possible to isolate between the two available phase current sensors, so both should to be checked to isolate the faulty sensor. Phase current sensors faults generate an oscillation which depends on the operating point, so in case of fixed threshold, the sensitivity for the same threshold is different depending on the operating point. An adaptive threshold to maintain the same sensibility is recommended, based on current components, estimated torque and motor speed. Residual envelope and fault relation is obtained by fault injection. The oscillation extraction is subject to motor electrical frequency estimation and bandwidth around it. The extraction filter should be redesign in case of a change in the execution period.

The FDI algorithm for speed sensors is the least demanding solution in terms of computational cost for traction drives where more than one speed measurement is available.

The main contribution of this work is the definition of an Integral Sensor Fault Detection and Isolation for a Railway traction drive, in opposite to most of the research works, which focus on only one or two kind of sensors. Moreover, it has been validated in a real Railway traction control unit, whereas the previous works have been validated in test benches without commercial control units, in rapid control prototyping devices.

Further research should be done with regard to fault severity estimation and fault reconstruction, in combination with information coming from other available tools in industry as FMEA. Fault injection and performance analysis under faults can provide information for an enhanced FMEA. This enhanced FMEA combines with FDI approaches, can provide reliable fault severity estimation. Furthermore, an adaptive threshold automation should be developed to optimize the sensibility of the detection and robustness for the different operating points of the motor.

## 9. Conclusions

This article has presented an Integral Sensor Fault Detection and Isolation for a Railway traction drive. The research aim was to implement an early fault detection in sensors, which allows improving the availability of traction drives. Taking into account that the strategy has to be executed by an embedded commercial traction control unit, low computational cost FDI approaches have been selected, due to commercial traction control unit limitations. Moreover, the use of easy to tune FDI algorithms for each application is a key point to obtain a successful industrial acceptance. The FDI approaches presented here, as well as the proposed Integral Sensor Fault Detection and Isolation, can be adapted to electric drives in other applications.

## Figures and Tables

**Table 1:** Summary of sensors in the Railway unit.

Sensor	Description
*v_cat_*	Catenary voltage sensors
*i_cat_*	Catenary current sensor
*i_ret_*	Return current to catenary sensor
*v_bus_*	DC-link voltage sensor
*i_crw_*	Braking unit current sensor
*i_u,v_*	Motor phase current sensors
*Encoder 1,2*	Motor speed sensors

**Table 2:** Diagnosability analysis for sensor faults for a time window of two samples.

	**Detectable**	*f_icat_*	*f_vbus_*	*f_vcat_*	*f_icrw_*	*f_inv_*
*f_icat_*	x	0	0	x	x	x
*f_vbus_*	x	0	0	0	x	x
*f_vcat_*	x	0	0	0	x	x
*f_icrw_*	x	0	0	x	0	0
*f_inv_*	x	0	0	x	0	0

**Table 3:** Likelihood ratio calculation for 10 V and 20 V offset fault in different scenarios.

$v_{bus}$ Measured Scenario	$s_{fault\;free}^{fault\;10\;V}$	$s_{fault\;free}^{fault\;20\;V}$
Fault-free	−45.73	−191.23
Offset fault 10 V	50.57	1.41
Offset fault 15 V	100.33	100.98
Offset fault 20 V	154.13	208.66

**Table 4:** Combination of indicators for speed sensor fault isolation.

Flag	Fault in Sensor $w_{m1}$	Fault in Sensor $w_{m2}$	Fault in Sensor $v_{train}$
$f_{1}$	1	0	1
$f_{2}$	0	1	1
$f_{12}$	1	1	0
